# The influence of observers’ sex on attention-demanding performance depends on performers’ sex

**DOI:** 10.3389/fpsyg.2015.01217

**Published:** 2015-08-31

**Authors:** Lijun Wang, Jinfeng Tan, Jiangtao Chen, Antao Chen

**Affiliations:** Key Laboratory of Cognition and Personality of Ministry of Education, School of Psychology, Southwest University, Chongqing, China

**Keywords:** post-error slowing (PES), the same-sex interaction, the opposite-sex interaction, sex difference, color flanker task

## Abstract

Post-error slowing (PES) indicates the slower responses after errors than after correct responses. Prior studies mainly focus on how the observation errors influence one own’s performance, there is no study investigating how other’s monitoring influence one own’s performance. Additionally, the issue that whether social context influences the PES effect differently for females and males is still unclear. To address aforementioned issues, we required the participants to interact with a same-sex or opposite-sex partner to complete a color flanker task together (they sat next to each other, Experiment 1). One was the performer (perform the flanker task), and the other was the observer (monitor the error responses of performer). They alternated their roles in two successive blocks. To further verify the role of the interaction context, a control experiment was conducted in the individual context (Experiment 2). The results revealed that (1) larger PES effect was observed in females than in males in the interaction context; (2) the sex difference of PES effect mainly benefited from the opposite-sex interaction; (3) larger PES effect was observed in the interaction context than in the individual context; (4) females’ performance was influenced after an interaction with a same-sex or opposite-sex partner, whereas males’ performance was merely influenced after an interaction with an opposite-sex partner. Taken together, these findings may suggest that (1) interaction context modulates the PES effect differently for females and males; (2) females are more susceptible to social information and hence more effective to adjust the post-error behaviors.

## Introduction

Cognitive control as a hallmark of cognitive flexibility plays an important role in monitoring and adjusting to the diverse environments and situations. An important aspect of cognitive control is the ability to detect and adjust error behaviors. Following error responses, three types of behavioral adjustments have been reported in the post-error trials (Danielmeier and Ullsperger, 2011; Ullsperger et al., 2014): post-error slowing (PES), post-error improvement in accuracy (PIA), and post-error reduction of interference (PERI). However, among these adjustments, the slowing in reaction time (RT) is the most stable phenomenon in the post-error trials. PES describes the prolonged RT in correct trials following errors (eC trial) compared to RT in correct trials following correct trials (cC trial; see Danielmeier and Ullsperger, 2011, for a review; Rabbitt, 1966; Laming, 1979; Dutilh et al., 2012; Wang et al., 2015).

When we perform a task with another person together, monitoring and learning from other’s actions is essential to efficiently adapt our behavior and to optimize task performance. In recent years, considerable evidences have demonstrated that the individual can adjust one own’s performance according to the error information of the person one is interacting with, as reflected in slower RT (Castellar et al., 2011; de Bruijn et al., 2011; Wang et al., 2015). Moreover, several studies have extended above finding by showing the difference in the slowing following observation errors depending on the social context (i.e., cooperative and competitive contexts; Castellar et al., 2011; de Bruijn et al., 2011). Research by de Bruijn et al. (2011) has found that participants slow down following observation errors only in the cooperation context by employing a social speeded choice RT task. Therefore, these studies suggest that the PES effect can be modulated by the social factor. However, it remains unknown whether and how other’s observation influence one own’s performance.

Additionally, the individual difference in cognitive control has aroused the researchers’ great interest (e.g., Hester et al., 2004; Garcia-Garcia et al., 2008; Li et al., 2009; Stoet, 2010; Kret and De Gelder, 2012; Wang et al., 2014). In particular, sex differences in cognitive monitoring have been documented within numerous literatures. For example, females are more distracted by the task-irrelevant information in the conflict-related tasks such as the flanker task (e.g., Bayliss et al., 2005; Garcia-Garcia et al., 2008; Stoet, 2010; Clayson et al., 2011). In a related vein, females have stronger inhibition ability to withhold inappropriate behaviors in the cognitive tasks such as the stop-signal task and the odd-ball task (e.g., Yuan et al., 2008; Li et al., 2009). However, although the ability of error monitoring is stronger in females, the adjustment following errors is not different between sexes (e.g., Li et al., 2009; Larson et al., 2011). It is worth noticing that heterosexual males’ cognitive performance is impaired after an interaction with one of female participants (Karremans et al., 2009), even when they merely anticipate an interaction with one female (Nauts et al., 2012). However, this cognitive impairment is not observed in females. To our knowledge, there have been no studies exploring the sex difference of the PES effect in the social context. Thus, it is appealing to investigate whether social contexts modulate the PES effect in a different way or to a different degree for females and males.

In the present study, we utilized two experiments to examine aforementioned issues by employing a modified flanker task (Eriksen and Eriksen, 1974). For the flanker task, Participants are required to respond to the central target while ignoring the flankers that may have the same response with the target (congruent trial) or an different response with the target (incongruent trial). Experiment 1 examined whether behavior was adjusted differently for females and males during social interactions. In this Experiment, the participants were instructed to interact with the other participant with same-sex or opposite-sex to execute a color flanker task (they sat next to each other). Two participants worked as a team, one of them was responsible for performing the task (performer), and the other was responsible for monitoring the performer’ responses (observer; cf. Koban et al., 2010). When the performer committed errors, the observer needed to press the spacebar in another keyboard to warn the performer. After completing one block, two participants altered their roles. In addition, to ensure the task involvement of the participants acting the observer, before the experiment, participants were informed that they would be rewarded as a team (Koban et al., 2010; Castellar et al., 2011; Dutilh et al., 2012). If both of them had good performance (more accurate than the other pairs not only performing the task but also counting the other’s errors), they would be paid extra bonus (Castellar et al., 2011). To further explore whether the social interactions influenced differently the behavioral adjustment for females and males, the control Experiment 2 was performed by the individuals alone.

## Experiment 1

In this experiment, two participants with same-sex or opposite-sex were instructed to sit next to each other and complete the task together. Fukushima and Hiraki (2006) instruct participants to play a competitive two-person gambling game and find that females treat the loss of opponent the similar as one own’s. This finding may suggest females are more sensitive to socio-negative events such as unpleasant events or monetary loss. In addition, prior studies have affirmed that the error monitoring of females is stronger (e.g., Li et al., 2009; Larson et al., 2011). Based on these studies, we predict that the PES effect of females should be larger than males in the interaction context. Otherwise, the interaction context modulates the PES effect equally for the females and males.

### Materials and Methods

#### Participants

Ninety-four healthy participants (47 females, all right-handed with normal color perception, and normal or corrected-to-normal vision, aged 19–30 years) took part in the experiment for payment. 44 participants (22 females) were assigned to the same-sex interaction and 50 participants (25 females) to the opposite-sex interaction. They were tested in pairs. All participants provided written informed consent and all of them were naive to the purpose of the experiment. Data from six participants (three pairs) were discarded, one pair (two females) in the same-sex interaction misunderstood the instruction and two pairs in the opposite-sex interaction lacked enough error trials. As suggested by a number of researchers (e.g., Olvet and Hajcak, 2009; Larson et al., 2010; Picton et al., 2012), between 6 and 14 trials are required for a reliable error processing. Thus, only those who had at least 15 trials for each condition were included in the analyses. Finally, data from 88 (43 females) participants were used for analyzing. Participants were free from neurological diseases and reported no history of psychoactive medication use. Approval of the study was made by the Human Research Ethics Committee of the Southwest University of China.

#### Apparatus and Stimuli

The experiment was conducted using E-Prime software (Psychology Software Tools, Inc. Pittsburgh, PA, USA) and run on a 17-inch monitor of a Dell computer (with a refresh rate of 85 Hz and a resolution of 1024 × 768). Participants were seated in a comfortable chair in a sound-attenuated chamber at a distance of approximately 60 cm away from the screen.

Eight color filled rounds (red, green, yellow, blue, orange, cyan, purple, and tawny) were employed to constitute the color flanker task, which was displayed on the gray background. The RGB values for stimulus colors were 255, 0, 0 (red); 0, 255, 0 (green); 255, 255, 0 (yellow); 0, 0, 255 (blue); 230, 110, 10 (orange); 0, 150, 80 (cyan); 110, 50, 160 (purple), and 150, 140, 80 (tawny), respectively. In each trial, a line of five color rounds was presented in the center of the screen. The central color round was the target and the remaining color rounds were the flankers. The 2-1 mapping rules (Zhang and Kornblum, 1998; De Houwer, 2003; Chen et al., 2011) was adopted to achieve enough error trials for the reliable analysis of the PES effect. When the target and the flankers were mapped onto the same response key, the trials were defined as congruent trials; when the target and the flankers were mapped onto different response keys, the trials were defined as incongruent trials. Congruent and incongruent trials were pseudo-randomly sequenced with equal frequency. Participants were instructed to respond to the central color round as quickly and correctly as possible on horizontally-arranged number keys on a standard keyboard. The stimulus-response mappings corresponding to the color rounds were counterbalance across the participants. For half of the participants, red and tawny rounds were mapped to the one key (left middle finger), cyan and orange rounds were mapped to the two key (left index finger), yellow and purple rounds were mapped to the nine key (right index finger), and blue and green rounds were mapped to the 0 key (right middle figure). And for the other half of the participants, red and tawny rounds were mapped to the 0 key (right middle figure), cyan and orange rounds were mapped to the nine key (right index finger), yellow and purple rounds were mapped to the two key (left index finger), and blue and green rounds were mapped to the one key (left middle finger).

#### Experimental Design

The sex differences in the PES effect were examined via a 2 by 2 by 2 mixed design, in which response type (eC and cC) as the within-subjects factor, sex of performer (female and male) and sex of observer (female and male) as the between-subjects factors. Before the formal experiment, participants first performed two practice blocks of 30 trials to get familiarized with the task and to reduce learning effects. The formal experiment consisted of six successive blocks (120 trial each, 720 trials in all), with a 30 s break between blocks, lasting approximately 60 min.

#### Experiment Procedure

**FIGURE 1:**
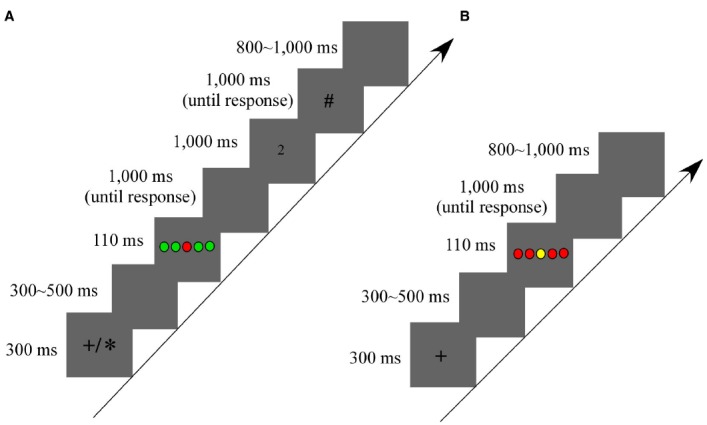
**Schematic illustration of the procedures. (A)** The experimental setup of the interaction context used in Experiment 1. **(B)** The experimental setup of the individual context used in Experiment 2. Notably, in the interaction context, two participants were arranged to sit next to someone of the same sex or the opposite sex and to complete the task together. One was responsible for performing the flanker task (performer), and the other was responsible for observing the partner’s performance (observer). They exchanged their roles after completing one block. The cross fixation (+) cued participant A as the performer, and the asterisk (*) cued participant B as the performer. In the individual context, the participant completed the task alone.

Figure 1A displays the timing of one trial in the interaction context. Each trial started with a 300 ms fixation (+/*). The cross (+) fixation cued participant A as a performer, while the asterisk (*) fixation cued participant B as a performer. After completing one block, their roles exchanged. When the fixation disappeared, a blank screen was presented in a random interval for 300–500 ms. Then, an array of five color rounds presented in the center of the screen for 110 ms, followed by a 1,000 ms blank screen. Participants needed to respond to the central target in this interval, with a maximal time limitation of 1,000 ms. After a response was made, the blank screen immediately disappeared and the response of the performer (one of the four response keys) presented in the screen for 1,000 ms, which would help the observers to realize the correctness of key-press of their partners. Next, the number sign (#) presented the screen for 1,000 ms. Here, the observers were instructed to press the spacebar when they observed their partners made wrong responses (terminated after the spacebar was pressed within this interval). When the performers’ responses were correct, the observers did not need to respond, and the number sign would last for 1,000 ms. Finally, a 800–1,000 ms interval was presented (interval varied randomly).

### Results

**FIGURE 2:**
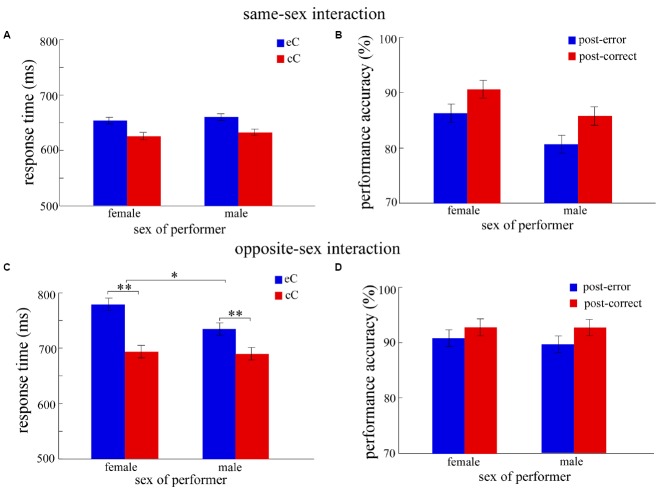
**The results of the interaction context in Experiment 1.** To examine whether the sex of observer influence the performance of participants differently for females and males, we conducted the ANOVA in the same-sex interaction and the opposite-sex interaction respectively, with response type (eC and cC) as a within-subjects factor and sex of performer (female and male) as a between-subjects factor. **(A,B)** show the results of RT and accuracy in the same-sex interaction respectively; **(C,D)** show the results of RT and accuracy in the opposite-sex interaction respectively. Blue bars indicate the mean RT of post-error correct responses (eC, **A,C**) or the performance accuracy of post-error trials **(B,D)**, and red bars indicate the mean RT of post-correct correct responses (cC, **A,C**) or the performance accuracy of post-correct trials **(B,D)**. Error bars denote standard error, *p < 0.05, **p < 0.01.

**FIGURE 3:**
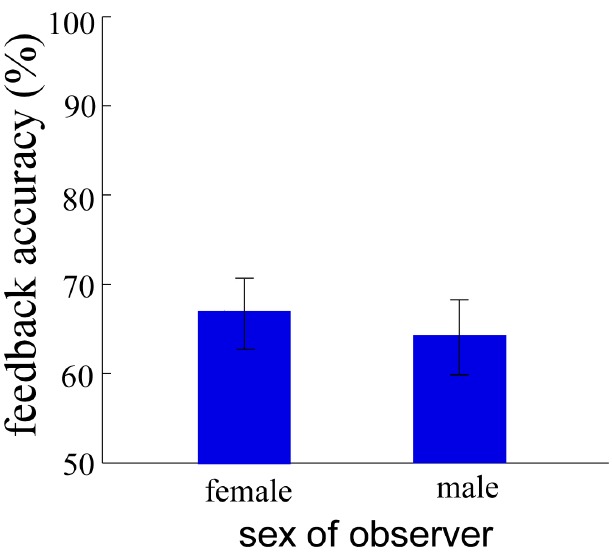
**The result of feedback accuracy in Experiment 1.** Error bars denote standard error.

For the RT analyses, the error trials, and trials that the RTs were shorter than 150 ms and longer than 1,000 ms were eliminated. In total, 11% of all data was excluded. The results for PES, post-error accuracy and feedback accuracy in the interaction context as a function of experimental factors are shown in Figures 2 and 3.

#### Post-Error Slowing

For the PES effect, a three-way analysis of variance (ANOVA) was conducted, with response type (eC and cC) as the within-subjects factor, sex of performer (female and male) and sex of observer (female and male) as the between-subjects factors. The Greenhouse–Geisser correction was employed where appropriate. The results revealed that the main effect of response type was significant [*F*_(1,84)_ = 102.36, *p* < 0.001], with slower response in eC trials. However, neither the main effect of sex of performer nor the main effect of sex of observer was significant [all *F*_(1,84)_ < 1]. Nevertheless, the three-way interaction between response type, sex of performer and sex of observer was significant, *F*_(1,84)_ = 15.55, *p* < 0.001. *Post hoc* tests revealed that females worked with another female observer, the mean RT of eC trials (654 ± 19 ms) was significantly larger than that of cC trials (625 ± 17 ms), *F*_(1,84)_ = 8.81, *p* < 0.05. Females worked with another male observer, the mean RT of eC trials (779 ± 18 ms) was significantly larger than that of cC trials (693 ± 15.7 ms), *F*_(1,84)_ = 89.55, *p* < 0.001. Males worked with another male observer, the mean RT of eC trials (661 ± 18 ms) was significantly larger than that of cC trials (632 ± 16 ms), *F*_(1,84)_ = 9.43, *p* < 0.05. Males worked with another female observer, the mean RT of eC trials (735 ± 18 ms) was significantly larger than that of cC trials (690 ± 15.7 ms), *F*_(1,84)_ = 24.38, *p* < 0.001. Moreover, the two-way interaction between response type and sex of performer was significant [*F*_(1,84)_ = 4.98, *p* < 0.05], showing the magnitude of the PES effect was significantly larger for females than for males. *Post hoc* tests revealed that the mean RT of eC trials (717 ± 13 ms) was significantly larger than that of cC trials (659 ± 11 ms) for females [*F*_(1,84)_ = 74.38, *p* < 0.001], suggesting the PES effect (RT_*eC*–*cC*_ = 57.70 ms) occurred in females. Likewise, the mean RT of eC trials (698 ± 13 ms) was significantly larger than that of cC trials (661 ± 11 ms) for males [*F*_(1,84)_ = 31.89, *p* < 0.001], suggesting the PES effect (RT_*eC*–*cC*_ = 36.85 ms) occurred in males. The two-way interaction between gender of performer and gender of observer was significant [*F*_(1,84)_ = 23.63, *p* < 0.001]. *Post hoc* tests revealed that the mean RT was significantly slower for the opposite-sex interaction (736 ± 16 ms) than for the same-sex interaction (640 ± 17 ms) in females, *F*_(1,84)_ = 16.32, *p* < 0.001. Likewise, the mean RT was significantly slower for the opposite-sex interaction (712 ± 16 ms) than for the same-sex interaction (647 ± 17 ms) in males, *F*_(1,84)_ = 7.95, *p* < 0.01.

Further, to make clear whether the sex difference of PES effect was influenced by the sex of observer, we analyzed the same-sex interaction and the opposite-sex interaction respectively. Two two-way ANOVAs were conducted, with response type as the within-subjects factor and the sex of performer as the between-subjects factor. In the same-sex interaction (Figure 2A), the results revealed that the main effect of response type was significant, *F*_(1,40)_ = 45.29, *p* < 0.001. However, neither the main effect of sex of performer nor the two-way interaction was significant, all *F*_(1,40)_ < 1. In the opposite-sex interaction (Figure 2C), the results revealed that the main effect of response type was significant, *F*_(1,44)_ = 67.17, *p* < 0.001. However, the main effect of sex of performer was not significant, *F*_(1,44)_ < 1. Importantly, the two-way interaction reached a significant level, *F*_(1,44)_ = 6.63, *p* < 0.05, showing the magnitude of the PES effect was significantly larger for females than for males. *Post hoc* tests revealed that the mean RT of eC trials (779 ± 22.7 ms) was significantly larger than that of cC trials (693 ± 19 ms) for females [*F*_(1,44)_ = 58.01, *p* < 0.001], suggesting the PES effect (RT_*eC*–*cC*_ = 86.35 ms) occurred in females. Likewise, the mean RT of eC trials (735 ± 22.7 ms) was significantly larger than that of cC trials (690 ± 19 ms) for males [*F*_(1,44)_ = 15.79, *p* < 0.001], suggesting the PES effect (RT_*eC*–*cC*_ = 86.35 ms) occurred in males.

#### Post-Error Accuracy

Post-error accuracy analysis focused on the performance accuracy of trials following errors when participants acted as performers. For the post-error accuracy analysis, a three-way ANOVA was conducted, with response type (post-error accuracy and post-correct accuracy) as the within-subjects factor, and sex of performer (female and male) and sex of observer (female and male) as the between-subjects factors. The Greenhouse–Geisser correction was employed where appropriate. The results revealed that the main effect of response type was significant [*F*_(1,84)_ = 22.99, *p* < 0.001], with more errors after error responses. The main effect of sex of performer was significant [*F*_(1,84)_ = 3.86, *p* < 0.05], with higher performance accuracy in females. Moreover, the interaction between sex of performer and sex of observer was significant [*F*_(1,84)_ = 14.90, *p* < 0.001]. *Post hoc* tests revealed that the performance accuracy was higher for males working with the opposite-sex partner (92 ± 1.5%) than those working with the same-sex partner (83 ± 1.5%), *F*_(1,84)_ = 14.64, *p* < 0.001. However, no any other significant results was found, all *F*_(1,84)_ < 2.20.

As the analysis of PES effect, we analyzed respectively the same-sex interaction and the opposite-sex interaction for the post-error accuracy. Two two-way ANOVAs were conducted, with response type as the within-subjects factor and the sex of performer as the between-subjects factor. In the same-sex interaction (Figure 2B), the results revealed that the main effects of response type [*F*_(1,40)_ = 17.07, *p* < 0.001] and sex of performer [*F*_(1,40)_ = 4.50, *p* < 0.05] were significant. However, the two-way interaction was not significant, *F*_(1,40)_ < 1. In the opposite-sex interaction (Figure 2D), the results revealed that the main effect of response type, *F*_(1,44)_ = 6.58, *p* < 0.05. However, neither the main effect of sex of performer nor the two-way interaction was significant, all *F*_(1,44)_ < 1.

#### Feedback Accuracy

Feedback accuracy analysis indicated the correct feedback rates of participants when they acted as the observer. An individual-sample *t*-test was conducted (Figure 3) to investigate the sex difference in feedback accuracy. The results revealed that feedback accuracy was not significantly different between females (67 ± 28%) and males (64 ± 27%), *t*_(86)_ = 0.40, *p* > 0.1.

### Discussion

Pairs of participants performing the color flanker task together revealed that the PES effect was observed for both females and males. The result pattern of PES effect manifested that the mean RT of eC trials was significantly prolonged compared to that of cC trials. Moreover, the PES effect was observed significantly larger in females than in males, suggesting sex difference exists in the post-error behavioral adjustment in the interaction context. More importantly, the sex difference of the PES effect only appeared in the opposite-sex interaction, but not in the same-sex interaction. Additionally, the mean RT was significantly slower for the opposite-sex interaction than for the same-sex interaction in both females and males. These findings may suggest that an interaction with unacquainted opposite-sex others requires more cognitive control to complete current task.

Results for the accuracy of post-error response were revealed that higher performance accuracy in females than in males. It might be that females were more conscientious when they worked with a partner and therefore put more emphasis on accuracy than speed after an error. This may explain why magnitude of the PES effect was higher for females in the interaction context. Additionally, the post-error accuracy of male participants was higher in the opposite-sex interaction relative to the same-sex interaction. This result may suggest that males’ cognitive functioning is influenced by the sex of partner, and in a way of improvement. However, for the feedback accuracy, the performance between sexes was comparable, suggesting that the task involvement of females and males did not different when they acted as the observers.

## Experiment 2

Since previous studies have affirmed that there is no sex difference in the PES effect under the individual context (Hester et al., 2004; Li et al., 2009; Larson et al., 2011). To compare with the findings of previous studies and further verify the role of interaction context in sex difference of the PES effect, we conducted Experiment 2 without the interaction context. For this purpose, we arranged the participants to execute the color flanker task alone. In addition, the instruction that two of participants were as a team to be rewarded according to their joint performances was eliminated.

### Materials and Methods

#### Participants

Forty-six healthy participants (24 females, all right-handed except for one female and one male who were left-handed, all with normal color perception, and normal or corrected-to-normal vision, aged 19–24 years) took part in the experiment for payment. All participants provided written informed consent and all of them were naive to the purpose of the experiment. Data from two females and one male were discarded because of committing too many errors (performance accuracy < 75%). Finally, data from forty-three (22 females) participants were used for analyzing. Approval of the study was made by the Human Research Ethics Committee of the Southwest University of China.

#### Apparatus, Stimuli, and Experimental Design

The apparatus and stimuli utilized in Experiment 2 were the same as in Experiment 1. However, in Experiment 2, the participants were required to complete the task alone. In this case, a 2 by 2 mixed design was adopted, with the response type (eC and cC) as a within-subjects factor and sex of performer (female and male) as a between-subjects factor. Before the experiment, participants were instructed to complete the task as quickly and correctly as possible. The experiment consisted of four successive blocks (120 trials in one block, 480 trials in all), with a 30 s break between blocks, lasting approximately 40 min.

#### Experiment Procedure

Figure 1B displays the experimental setup and time parameters of one trial in the individual context. Each trial started with a cross fixation for 300 ms, followed by a 300–500 ms random blank screen. Then, an array of five color rounds presented in the center of the screen for 110 ms, followed by a 1,000 ms blank screen. Participants needed to respond to the central target in this interval, with a maximal time limitation of 1,000 ms. Finally, a random blank screen was presented for 800–1,000 ms.

### Results

**FIGURE 4:**
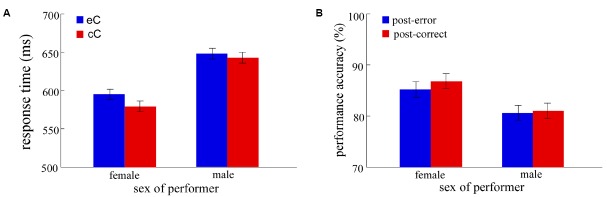
**The results of individual context in Experiment 2. (A,B)** show the results of RT and accuracy as a function of response type and sex of performer, respectively. Blue bars indicate the mean RT of post-error correct responses (eC, **A**) or the performance accuracy of post-error trials **(B)**, and red bars indicate the mean RT of post-correct correct responses (cC, **A**) or the performance accuracy of post-correct trials **(B)**. Error bars denote standard error.

**FIGURE 5:**
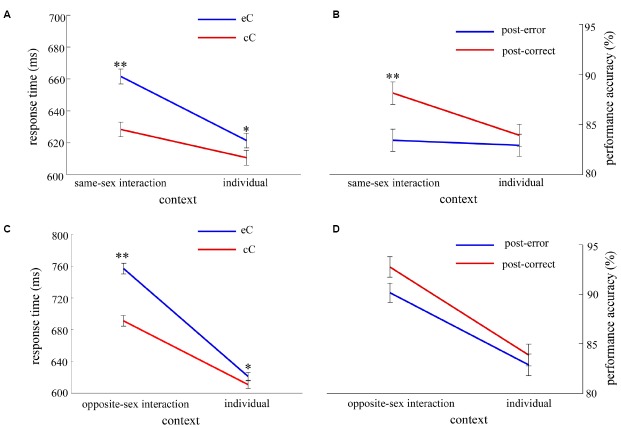
**The contrast results as a function of response type (eC and cC) and context (same-sex interaction/opposite-sex interaction and individual). (A,B)** show the contrast results of RT and accuracy between the same-sex interaction and individual contexts. **(C,D)** show the contrast results of RT and accuracy between the opposite-sex interaction and individual contexts. Blue lines indicate the mean RT of post-error correct responses (eC, **A,C**) or the performance accuracy of post-error trials **(B,D)**, and red lines indicate the mean RT of post-correct correct responses (cC, **A,C**) or the performance accuracy of post-correct trials **(B,D)**. Error bars denote standard error, *p < 0.05, **p < 0.01.

**FIGURE 6:**
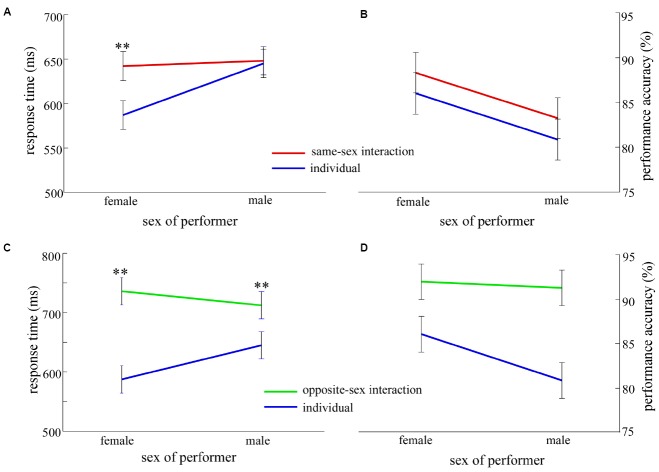
**The contrast results as a function of context (same-sex interaction/opposite-sex interaction and individual) and sex of performer (female and male). (A,B)** show the contrast results of RT and accuracy between the same-sex interaction and individual contexts. **(C,D)** show the contrast results of RT and accuracy between the opposite-sex interaction and individual contexts. Red lines indicate the performance in the same-sex interaction context, green lines indicate the performance in the opposite-sex interaction context, and blue lines indicate the performance in the individual context. Error bars denote standard error, *p < 0.01.

For the RT analyses, the error trials, and trials that the RTs were shorter than 150 ms and longer than 1,000 ms were eliminated. In total, 16.5% of all trials were excluded from RT analysis. The results for PES effect and performance accuracy in the individual context as a function of experimental factors are shown in Figure 4. The results of contrast analysis between interaction and individual contexts are shown in Figures 5 and 6.

#### Post-Error Slowing

For the PES effect, a two-way ANOVA was conducted, with response type (eC and cC) as a within-subjects factor, and sex of performer (female and male) as a between-subjects factor (Figure 4A). The results revealed that the mean RT of eC trials was significantly slower than that of cC trials [*F*_(1,41)_ = 4.77, *p* < 0.05], and females responded significantly faster than males [*F*_(1,41)_ = 12.43, *p* = 0.001]. However, the two-way interaction was not significant [*F*_(1,41)_ = 1.02, *p* > 0.1].

#### Post-Error Accuracy

For the post-error accuracy, a two-way ANOVA was conducted, with response type (post-error accuracy and post-correct accuracy) as a within-subjects factor, and sex of performer (female and male) as a between-subjects factor (Figure 4B). The results revealed that the main effect of sex of performer was significant [*F*_(1,41)_ = 5.55, *p* < 0.05], with higher accuracy in females than in males. However, neither the main effect of response type nor the two-way interaction was significant [all *F*_(1,41)_ < 1].

#### Contrast Analysis of Post-Error Effects Between the Same-Sex Interaction and Individual Contexts

Two three-way ANOVAs were respectively conducted with the following variables for the mean RT and accuracy to compare the results between same-sex interaction and individual contexts: response type (eC and cC for PES effect analysis; post-error accuracy and post-correct accuracy for accuracy analysis) as the within-subjects factor, sex of performer (female and male) and context (same-sex interaction and individual) as the between-subjects factors.

For RT, the results of ANOVA revealed that the main effects of response type [*F*_(1,81)_ = 44.56, *p* < 0.001], sex of performer [*F*_(1,81)_ = 8.39, *p* < 0.01], and context [*F*_(1,81)_ = 6.78, *p* < 0.05] were significant. Moreover, the two-way interaction between response type and context was significant (Figure 5A), *F*_(1,81)_ = 11.52, *p* < 0.01. *Post hoc* tests revealed that the mean RT of eC trials was significantly slower in the same-sex interaction context (662 ± 8 ms) than in the individual context [621 ± 8 ms; *F*_(1,81)_ = 12.04, *p* < 0.01]. However, this was not case for cC trials [*F*_(1,81)_ < 0.15]. The two-way interaction between sex of performer and context was significant (Figure 6A), *F*_(1,81)_ = 5.17, *p* < 0.05. *Post hoc* tests revealed that the mean RT was significantly slower in the same-sex interaction context (642 ± 11 ms) than in the individual context (587 ± 11 ms) only for females [*F*_(1,81)_ = 11.75, *p* < 0.05]. However, neither the two-way interaction between response type and sex of performer nor the three-way interaction reached a significant level, all *F*_(1,81)_ < 1.

For accuracy, the results revealed that the main effects of response type [*F*_(1,81)_ = 13.45, *p* < 0.001] and sex of performer [*F*_(1,81)_ = 9.98, *p* < 0.01] were significant. Moreover, the two-way interaction between response type and context was significant (Figure 5B), *F*_(1,81)_ = 5.82, *p* < 0.05. *Post hoc* tests revealed that the higher post-correct accuracy was observed in the same-sex interaction context (88 ± 1%) relative to the individual context (84 ± 1%), *F*_(1,81)_ = 9.03, *p* < 0.01, but this was not case for post-error accuracy, *F*_(1,81)_ < 1. However, all other effects were not significant, all *F*_(1,81)_ < 2.1.

#### Contrast Analysis of Post-Error Effects Between the Opposite-Sex Interaction and Individual Contexts

The same analysis was conducted as the contrast analysis of post-error effects between the same-sex interaction and individual contexts. For RT, the results of ANOVA revealed that the main effects of response type [*F*_(1,85)_ = 63.77, *p* < 0.001] and context [*F*_(1,85)_ = 5.85, *p* < 0.05] were significant. Moreover, the two-way interaction between response type and sex of performer was significant, *F*_(1,85)_ = 7.17, *p* < 0.01, showing significantly larger PES effect in females than in males. *Post hoc* tests revealed that the mean RT of eC trials (687 ± 13 ms) was significantly slower than that of cC trials (636 ± 11 ms) in females, *F*_(1,85)_ = 57.55, *p* < 0.001, suggesting the PES effect (RT_*eC*–*cC*_ = 51.1 ms) occurred in females. For males, the mean RT of eC trials (691 ± 13 ms) was significantly slower than that of cC trials (666 ± 11 ms), *F*_(1,85)_ = 13.92, *p* < 0.001, suggesting the PES effect (RT_*eC*–*cC*_ = 25.44 ms) occurred in males. The two-way interaction between response type and context was significant (Figure 5C), *F*_(1,85)_ = 32.78, *p* < 0.001. The simple effect showed that the mean RT of eC trials (757 ± 16 ms) was significantly slower than that of cC trials (691 ± 13 ms) in the opposite-sex interaction context, *F*_(1,45)_ = 59.70, *p* < 0.001, suggesting the PES effect (RT_*eC*–*cC*_ = 65.7 ms) occurred in the opposite-sex interaction. Likewise, the mean RT of eC trials (621 ± 9 ms) was significantly slower than that of cC trials (610 ± 10 ms) in the individual context, *F*_(1,42)_ = 4.89, *p* < 0.05, suggesting the PES effect (RT_*eC*–*cC*_ = 10.8 ms) occurred in the individual context. The two-way interaction between sex of performer and context was significant (Figure 6C), *F*_(1,85)_ = 5.85, *p* < 0.05. *Post hoc* tests revealed that the mean RT was significantly slower in the opposite-sex interaction context (female: 736 ± 16 ms; male: 712 ± 16 ms) than in the individual context (female: 587 ± 16 ms; male: 645 ± 17 ms) for both females [*F*_(1,85)_ = 39.72, *p* < 0.001] and males [*F*_(1,85)_ = 8.0, *p* < 0.01]. However, no other effects was significant, all *F*_(1,85)_ < 2.7.

For accuracy, the results revealed that the main effects of response type [*F*_(1,85)_ = 6.03, *p* < 0.05], sex of performer [*F*_(1,85)_ = 4.39, *p* < 0.05], and context [*F*_(1,85)_ = 32.71, *p* < 0.001] were significant. However, all other effects were not significant, all *F*_(1,85)_ < 2.5.

### Discussion

Experiment 2 revealed that the two-way interaction (response type and sex of performer) was not significant, suggesting the PES effect was no difference between females and males in the individual context. This result was consistent with the findings of previous studies (Hester et al., 2004; Li et al., 2009; Larson et al., 2011).

To examine how the interaction context influenced the performance of participants, and whether modulated the behaviors of females and males in a different way, we conducted respectively the contrast analysis of the post-error effects for the same-sex interaction and individual contexts and for the opposite-sex interaction and individual contexts. The results revealed that no matter whether the partner was same-sex or opposite-sex, larger PES effect was observed in the interaction context than the individual context (Figures 5A,C). This confirms that the PES effect is modulated by the social context (Castellar et al., 2011; de Bruijn et al., 2011). Additionally, as shown in Figures 6A,B, for females, the mean RT was significantly slower in the interaction context including both the same-sex interaction and the opposite-sex interaction than in the individual context. However, for males, the slower RT only occurred in the opposite-sex interaction, relative to the individual context. These findings may suggest that females have the stronger sensibility of social information, and their cognitive performance can be influenced after an interaction with someone of the same sex or the opposite sex. However, males’ cognitive performance is only influenced after an interaction with someone of the opposite sex.

## General Discussion

The goals of present study were to investigate whether other’s observation could influence one own’s post-error adjustments, and whether the sex difference of PES effect occurred in the interaction context. Experiment 1, in which participants interacted with a same-sex or opposite-sex partner, demonstrated that the PES effect was significantly larger in females than in males. Moreover, this sex difference of PES effect merely occurred in the opposite-sex interaction, but not in the same-sex interaction. In Experiment 2, the participants were required to complete the task alone. The results revealed that the PES effect between sexes was comparable in the individual context. Additionally, the contrast analysis of post-error effects between the interaction and individual contexts revealed that the PES effect was significantly larger in the interaction context than in the individual context. More importantly, in the same-sex interaction, slower RT was merely observed in females, relative to the mean RT of individual context; whereas in the opposite-sex interaction, slower RT was observed both in females and males. Taken together, these findings may suggest that (1) the PES effect was modulated by the social context; (2) the sex of observer influenced the cognitive functioning of performer in a different way; (3) the females were more sensitive to the social information.

Paralleling with previous studies, the PES effect was comparable between females and males in the individual context (Li et al., 2009; Larson et al., 2011). However, in the interaction context, the PES magnitude was significantly larger in females than in males, suggesting that the sex difference of PES effect occurred in the interaction context. The possible reason is that females are more conscientious when they work with an observer and therefore put more emphasis on accuracy than speed after an error. In favor of this account, the performance accuracy was higher in females than in males. Alternative account is that females are expected to be friendlier than males in the society (Cross and Madson, 1997; Eagly and Wood, 1999). Gender stereotypes define females are more communal, conveying a concern for the welfare of others; whereas males are more agentic, conveying a concern for own outcomes over others’ outcomes (Conway et al., 1996; Witt and Wood, 2010). Gender stereotypes can influence behavior through self-regulatory processes, thus females may be more able to take more time to adjust their behaviors according to their partners’ feedback.

More importantly, the sex difference of PES effect merely appeared in the opposite-sex interaction, but not in the same-sex interaction (Figure 2). Additionally, the mean RT was significantly slower for participants working with an opposite-sex observer compared to participants working with a same-sex observer. These findings may suggest the sex of observer influences the participants’ cognitive performance. It is likely that people often have higher self-presentational concerns (such as making good impression) in the opposite-sex as compared to the same-sex interactions (Russell et al., 1986; Bruch et al., 1989; Karremans et al., 2009). Impression management generally requires relatively high levels of cognitive control (Vohs et al., 2005). In this case, relatively less cognitive recourses can be utilized for error adjustment in the opposite-sex interaction, and thus more time is taken to prepare the upcoming task. This might directly explain why the mean RT was slower in the opposite-sex interaction compared to the same-sex interaction. Besides, when participants work with the opposite-sex observers, females may be more nervous and more affected by the negative feedback. In this case, females will need more time to adjust the error. Moreover, females are more susceptible to the task-irrelevant information (Bayliss et al., 2005; Garcia-Garcia et al., 2008; Stoet, 2010). Therefore, for females, the adjustment following an error was prolonged in the opposite-sex interaction, relative to males.

Previous studies have indicated that the males’ cognitive functioning is damaged when they interact with the opposite-sex, behaving as slower RT on correct trials (Karremans et al., 2009; Nauts et al., 2012). Consistent with this finding, we found that slower RT in the opposite-sex interaction than in the same-sex interaction for males. However, for accuracy, higher performance accuracy was observed in the opposite-sex interaction compared to the same-sex interaction. This may suggest that males take a speed-accuracy tradeoff strategy to optimize their performance, resulting in the impairment in RT and the improvement in accuracy.

Furthermore, Castellar et al. (2011) have affirmed that the PES effect is modulated by social context (cooperation and competition), showing larger PES effect in the cooperation context than in the competition context. Current results were consistent with the above research that the interaction context did modulate the PES effect (Figures 5A,C). Larger PES effect was observed in both the same-sex interaction and the opposite-sex interaction, compared to the individual context. There were two possible reasons to cause current results. On the one hand, the mental alertness of participants increased in the interaction context since their performances were monitored both by internal error awareness and external error feedback, thus participants might adopt a more conservative strategy (slower and more accurate) to optimize the upcoming task in the interaction context. Previous studies have affirmed that the degree of mental alertness is a terrific predictor of individual differences in behavioral adjustments (e.g., Carp and Compton, 2009; Liu et al., 2013). On the other hand, according to the orienting account (Notebaert et al., 2009), the slowing occurs after errors because infrequent errors draw away attentional resources, and then participants need to take more time to reorient the subsequent task. In the present study, we instructed the partners to press the spacebar when they observed the error responses of the performers. Thus, the partners’ feedback might capture the performers’ attentional resources, resulting in slower response in eC trials in the interaction context.

Additionally, as shown in Figures 6A,C, the contrast analysis of post-error effects between interaction and individual contexts revealed that the mean RT was significantly slower for females in the interaction context (both the same-sex interaction and the opposite-sex interaction) than in the individual context, whereas the slower RT for males merely was observed in the opposite-sex interaction. These findings may suggest that the interaction context affects the performance of both females and males, but in a distinct way. Females are more sensitive to social information, and thus their performance can still be influenced after interacting with another female.

In conclusion, interaction context did modulate the PES effect, especially in females. With the color flanker task, we found larger PES effect in females than in males in the interaction context, but this was not the case for the individual context. Moreover, the sex difference of PES effect mainly benefited from the opposite-sex interaction. Additionally, the contrast analysis of post-error effects between interaction and individual contexts revealed that larger PES effect was observed in the interaction context than in the individual context. Particularly, females’ cognitive performance could be influenced by the interaction with a same-sex or opposite-sex observer; whereas males’ cognitive performance was merely influenced by the opposite-sex interaction. Integrating these findings might suggest that social interaction influence individuals’ cognitive performance, depending on whom the partner in the interaction.

Females were more susceptible to the social information and therefore more effective to post-error adjustment. In the future study, further evidences are required to obtain from neural dynamics to fully address these novel findings.

## Author Contributions

Substantial contributions to the conception or design of the work: LW; the acquisition, analysis, or interpretation of data for the work: LW, JT, JC; Drafting the work or revising it critically for important intellectual content: LW, AC; Final approval of the version to be published: AC; Agreement to be accountable for all aspects of the work in ensuring that questions related to the accuracy or integrity of any part of the work are appropriately investigated and resolved: AC.

### Conflict of Interest Statement

The authors declare that the research was conducted in the absence of any commercial or financial relationships that could be construed as a potential conflict of interest.

## Abbreviations

ANOVA: analysis of variance
cC trial: correct trials following correct trials
eC trial: correct trials following errors
PERI: post-error reduction of interference
PES: post-error slowing
PIA: post-error improvement in accuracy
RT: reaction time.
